# Pyrotinib combined with apatinib for targeting metastatic non-small cell lung cancer with *HER2* alterations: a prospective, open-label, single-arm phase 2 study (PATHER2)

**DOI:** 10.1186/s12916-022-02470-6

**Published:** 2022-08-29

**Authors:** Guangjian Yang, Haiyan Xu, Yaning Yang, Shuyang Zhang, Fei Xu, Xuezhi Hao, Junling Li, Puyuan Xing, Xingsheng Hu, Yutao Liu, Lin Wang, Lin Lin, Zhijie Wang, Jianchun Duan, Jie Wang, Yan Wang

**Affiliations:** 1 Department of Medical Oncology, National Cancer Center/National Clinical Research Center for Cancer/Cancer Hospital, Chinese Academy of Medical Sciences and Peking Union Medical College, No.17 Panjiayuan Street South, Chaoyang District, Beijing, 100021 China; 2 Department of Respiratory Medicine, Shandong Cancer Hospital and Institute, Shandong First Medical University and Shandong Academy of Medical Sciences, Jinan, 250117 Shandong China; 3 Department of Comprehensive Oncology, National Cancer Center/National Clinical Research Center for Cancer/Cancer Hospital, Chinese Academy of Medical Sciences and Peking Union Medical College, Beijing, 100021 China

**Keywords:** Pyrotinib, Apatinib, *HER2* mutation, *HER2* amplification, Non-small cell lung cancer

## Abstract

**Background:**

Although targeted agents have been gradually applied in the treatment of *HER2-*mutated non-small cell lung cancer (NSCLC) in recent years, patients’ therapeutic demands are far from being met. PATHER2 was the first phase 2 trial to explore the efficacy and safety of the *HER2*-targeted tyrosine kinase inhibitor (TKI) pyrotinib plus the antiangiogenic agent apatinib in previously treated *HER2*-altered metastatic NSCLC patients.

**Methods:**

*HER2*-mutated or *HER2*-amplified metastatic NSCLC patients who had failed at least first-line chemotherapy or *HER2*-targeted TKIs received oral pyrotinib 400 mg plus apatinib 250 mg once daily until disease progression, intolerable toxicity, or death. The primary endpoint was the investigator-assessed objective response rate (ORR).

**Results:**

Between March 2019 and December 2020, 33 patients were enrolled; 13 (39.4%) presented brain metastases, and 16 (48.5%) had received at least two lines of prior chemotherapy or *HER2*-targeted TKIs. As of September 20, 2021, the median follow-up duration was 11.3 (range, 3.5–26.0) months. The investigator-assessed ORR was 51.5% (17/33; 95% CI, 33.5 to 69.2%), and the disease control rate was 93.9% (31/33; 95% CI, 79.8 to 99.3%). The median duration of response, progression-free survival, and overall survival were 6.0 (95% CI, 4.4 to 8.6) months, 6.9 (95% CI, 5.8 to 8.5) months, and 14.8 (95% CI, 10.4 to 23.8) months, respectively. The most frequent grade ≥ 3 treatment-related adverse events included diarrhea (3.0%) and hypertension (9.1%). No treatment-related deaths were reported.

**Conclusions:**

Pyrotinib plus apatinib demonstrated promising antitumor activity and a manageable safety profile in *HER2*-mutated or *HER2*-amplified metastatic NSCLC patients.

**Trial registration:**

Chinese Clinical Trial Registry Identifier: ChiCTR1900021684.

## Background

Non-small cell lung cancer (NSCLC) is a heterogeneous malignant disease with a high degree of genomic diversity. During the past decade, the development of targeted therapies against specific molecular aberrations (e.g., epidermal growth factor receptor [*EGFR*] mutations, anaplastic lymphoma kinase [*ALK*] and *ROS1* fusions) has contributed to the improvement of patient prognosis, and targeted therapies have emerged as the standard of care for oncogene-driven NSCLC [1, 2]. In the era of genetic testing, more oncogenic driver genes, such as human epidermal growth factor receptor 2 (*HER2* or *ERBB2*), have been identified. *HER2* alterations, mainly manifested as protein overexpression, gene amplification, or gene mutation, occur in 2–4% of NSCLC patients [3, 4]. *HER2* alterations are commonly observed in female, never-smokers and lung adenocarcinoma patients, who have a higher probability of developing brain metastases than those without *HER2* alterations or other molecular mutations [3, 5, 6].

Unlike NSCLC patients with other common molecular aberrations, who have a remarkable response to tyrosine kinase inhibitors (TKIs), *HER2*-altered patients are relatively insensitive to *HER2*-targeted TKIs such as afatinib, neratinib, poziotinib, and dacomitinib, with an objective response rate (ORR) of less than 30% [7–10]. The standard of care for *HER2*-altered population is still chemotherapy or immunotherapy, also with limited benefit [11–13]. Currently, only two anti-*HER2* antibody–drug conjugates (ADCs), ado-trastuzumab emtansine (T-DM1) and trastuzumab deruxtecan (DS-8201), have shown encouraging results in *HER2*-mutated NSCLC, with an ORR of 44–55% [14, 15]. However, pulmonary toxicities may limit their application. Thus, more anti-*HER2* therapeutic approaches need to be explored.

Tumor angiogenesis is a critical feature of cancer pathogenesis, not only providing oxygen to the tumor but also providing an important pathway for the metastatic spread of cancer cells [16]. Antiangiogenic agents targeting the dominant angiogenic mediators vascular endothelial growth factor (*VEGF*) and *VEGF* receptor (*VEGFR*) could normalize pathologic tumor vasculature, modulate the tumor microenvironment, and suppress neovascularization [16, 17]. Previous evidence has shown that *EGFR*-TKIs could diminish *VEGF* expression and inhibit tumor angiogenesis; when combined with antiangiogenic agents, they facilitate substantial survival benefits in advanced NSCLC patients [18–20]. *VEGF* expression is also modulated by *HER2* signaling [21], and additional suppression of *VEGF/VEGFR* activity might potentiate the antitumor effects of *HER2* inhibitors.

Pyrotinib is an irreversible oral pan-*ErbB* TKI targeting *EGFR/HER1*, *HER2*, and *HER4*. In a phase II study, pyrotinib monotherapy as a second- or above-line treatment in *HER2*-mutant NSCLC patients showed an acceptable safety profile and promising antitumor activity, with an ORR of 30% [22]. Apatinib, an oral small-molecule TKI that selectively targets *VEGFR-2*, has displayed clinical benefit in patients with advanced NSCLC when combined with chemotherapy or *EGFR*-TKIs [19, 23]. In addition, apatinib exhibited synergistic antitumor effects with pyrotinib in vivo [24]. Herein, we conducted a single-arm phase 2 clinical study to explore the efficacy and safety of pyrotinib combined with apatinib in metastatic NSCLC patients harboring *HER2* mutations or amplification.

## Methods

### Study design and participants

PATHER2 is an open-label, single-arm, phase 2 clinical study (ChiCTR1900021684) conducted at National Cancer Center/Cancer Hospital, Chinese Academy of Medical Sciences (Beijing, China). Eligible patients were aged 18–70 years; had histologically or cytologically confirmed *HER2*-altered stage IV NSCLC (AJCC 8th edition); had at least one measurable lesion per the Response Evaluation Criteria in Solid Tumours, version 1.1 (RECIST 1.1); had received at least one kind of prior chemotherapy or anti-*HER2* TKIs for metastatic disease; had an Eastern Cooperative Oncology Group Performance Status (ECOG PS) of 0 or 1; and had adequate organ function. *HER2* alterations included *HER2* exon 20 insertion mutations, *HER2* missense mutations in the tyrosine kinase domain (TKD), and *HER2* amplification. Asymptomatic brain metastasis was permitted, except for meningeal metastasis. The key exclusion criteria included uncontrolled hypertension, significant gastrointestinal function abnormalities, a history of bleeding or the presence of clinically meaningful bleeding symptoms or definite bleeding tendency, an active and severe infection, and prior exposure to pyrotinib and/or apatinib.

This study was conducted in accordance with the Declaration of Helsinki and Good Clinical Practices and was approved by the Ethics Committee of National Cancer Center/Cancer Hospital, Chinese Academy of Medical Sciences. Written informed consent was obtained from each participant before study initiation.

### Procedures

All eligible patients were administered oral pyrotinib 400 mg and apatinib 250 mg once daily in a continuous 28-day cycle until disease progression, intolerable toxicity, or death. Dose reduction of pyrotinib was allowed according to adverse events, with a gradual reduction from 400 to 320 mg to 240 mg. In terms of apatinib, dose modification was not permitted, but dose interruption or discontinuation was allowed.

The initial tumor response was evaluated 4 weeks after the initiation of pyrotinib with apatinib and then every 8 weeks thereafter as per RECIST 1.1. Patients achieving a complete response (CR) or partial response (PR) had to be confirmed at least 4 weeks following the initial response. Computed tomography (CT) scans of the neck, chest, and abdomen were performed at baseline and then every 8 weeks until disease progression. Brain magnetic resonance imaging (MRI) and SPECT whole body bone scans were also performed at baseline and were not routinely taken unless metastasis of the central nervous system (CNS) or bone was clinically indicated. Adverse events (AEs) were collected from the onset of study treatment until 30 days after the last administration and were graded according to the National Cancer Institute-Common Toxicity Criteria for Adverse Events (NCI-CTCAE) version 5.0.

*HER2* alterations were documented by next generation sequencing (NGS) or amplification refractory mutation system polymerase chain reaction (ARMS-PCR) assay. DNA samples extracted from tumor tissues or plasma was used for *HER2* testing. Circulating tumor DNA from plasma was used only in cases of the inadequacy of tumor tissues. NGS testing was performed based on the Illumina sequencing system.

### Outcomes

The primary endpoint was investigator-assessed ORR, defined as the proportion of patients with a CR or PR as per RECIST 1.1. The secondary endpoints included progression-free survival (PFS, defined as the time from the initiation of study treatment to the first evidence of disease progression or death of any cause), disease control rate (DCR, defined as the proportion of patients with a CR, PR or stable disease [SD] as per RECIST 1.1), duration of response (DoR, defined as the time from the first documented response to the first evidence of disease progression or death of any cause), overall survival (OS, defined as the time from the initiation of study treatment to death of any cause), and safety.

### Statistical analysis

The sample size of this trial was calculated in accordance with Simon's optimal two-stage design, with a one-sided α error of 5% and a power of 80%. Ten evaluable patients were required in the first stage; if two or more of them achieved CR or PR, 19 additional evaluable patients were recruited in the second stage. An ORR of 10% was deemed unacceptable, whereas a 30% ORR was considered to be clinically promising and would merit further investigation. To account for a 10% dropout rate for unevaluable patients, a total of 33 patients were required.

Efficacy and safety were evaluated in patients undergoing at least one dose of study treatment. Patient characteristics and treatment-related adverse events (TRAEs) were summarized by descriptive statistics. The 95% confidence intervals (CI) for the ORR and DCR was calculated using the Clopper-Pearson method. The Kaplan–Meier method was performed to estimate PFS, DoR, and OS, and the 95% CI for median survival time and survival rate were calculated using the Brookmeyer-Crowley and complementary log–log method, respectively. Post hoc subgroup analyses for ORR and PFS were conducted on the basis of patient characteristics. Statistical analyses were performed by the SAS software, version 9.4 (SAS Institute). Forest plots were generated with the R software, version 4.3.1.

## Results

### Baseline characteristics

A total of 33 eligible patients with lung adenocarcinoma were enrolled from March 2019 to December 2020 in this study and received scheduled treatment with pyrotinib combined with apatinib. *HER2* status was determined by local or institutional laboratory tests in 32 patients (29 patients with tissues, 3 patients with plasma) by NGS and one patient with tissue by PCR in the institutional laboratory. The median age of the enrolled patients was 54 (range, 35–70) years. Twenty-four (72.7%) patients had an ECOG PS of 0, 13 (39.4%) presented brain metastases, and 16 (48.5%) had received at least two lines of prior chemotherapy or *HER2*-targeted TKIs. Of the 33 patients, 28 (84.8%) harbored *HER2* exon 20 insertions, with A775_G776insYVMA being the predominant insertion variant (60.6%). Three patients carried *HER2* TKD missense mutations, with one each of the G776V, R811L with Q820K and G727A mutations, and the remaining two patients harbored primary *HER2* amplification. The detailed baseline characteristics are listed in Table 1. As of September 20, 2021, the median treatment duration was 6.9 months (range, 0.9 to 17.3), with three patients (9.1%) still on treatment. The median follow-up duration was 11.3 (range, 3.5–26.0) months.

**Table 1:** Baseline characteristics of patients

Characteristic	*N* = 33
Median age, years (range)	54 (35–70)
≤ 60, *n* (%)	25 (75.8)
> 60, *n* (%)	8 (24.2)
Gender, *n* (%)	
Male	17 (51.5)
Female	16 (48.5)
Smoking history, *n* (%)	
Former/current	12 (36.4)
Never	21 (63.6)
ECOG PS, *n* (%)	
0	24 (72.7)
1	9 (27.3)
Brain metastases, *n* (%)	
Presence	13 (39.4)
Absence	20 (60.6)
No. of lines of prior systemic treatment, *n* (%)	
1	17 (51.5)
2	9 (27.3)
≥ 3	7 (21.2)
Median (range)	1 (1–5)
Prior Chemotherapy, *n* (%)	
Yes	24 (72.7)
No	9 (27.3)
Prior anti-*HER2* TKI therapy, *n* (%)	
Yes	17 (51.5)
No	16 (48.5)
*HER2* alterations, *n* (%)	
Exon 20 insertion mutations	28 (84.8)
A775_G776insYVMA Non-YVMA insertions*	20 (60.6) 8 (24.2)
TKD missense mutations**	3 (9.1)
Amplification	2 (6.1)

^*^Non-YVMA insertions included P780_Y781insGSP (*n* = 6), G776delinsVC (*n* = 1), and G776_V777delinsCVC (*n* = 1)

^**^TKD missense mutations included G776V (*n* = 1) and R811L with Q820K (*n* = 1) at exon 20, and G727A (*n* = 1) at exon 18

*ECOG PS* Eastern Cooperative Oncology Group Performance Status, *TKD* tyrosine kinase domain

### Clinical efficacy

**Fig. 1:**
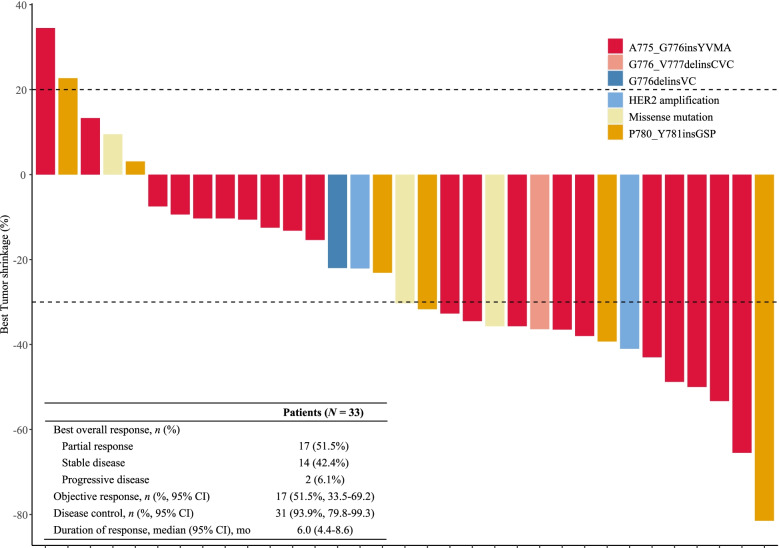
Waterfall plot of best response

**Fig. 2:**
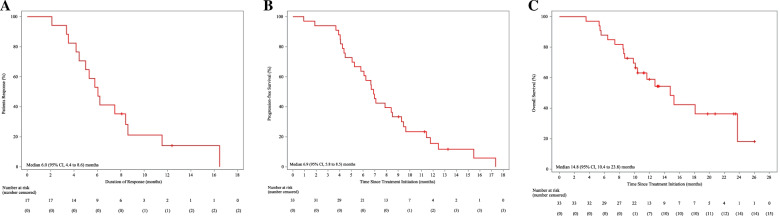
Kaplan–Meier estimates of the duration of response (**A**), progression-free survival (**B**), and overall survival (**C**)

Among the 33 evaluable patients, 17 (51.5%) achieved a PR, and 14 (42.4%) showed SD, with a confirmed investigator-assessed ORR of 51.5% (17/33; 95% CI, 33.5% to 69.2%) and a DCR of 93.9% (31/33; 95% CI, 79.8% to 99.3%). The independent review committee-assessed ORR was 48.5% (16/33, 95% CI 30.8% to 66.5%). In total, 84.8% (28/33) of patients experienced a decrease in target lesion size from baseline (Fig. 1). The median DoR was 6.0 (95% CI, 4.4 to 8.6) months, and the median PFS was 6.9 (95% CI, 5.8 to 8.5) months (Fig. 2A, B). At the time of data cut-off, 18 (54.5%) of 33 patients had died, with a median OS of 14.8 (95% CI, 10.4 to 23.8) months (Fig. 2C) and a 1-year overall survival rate of 58.9% (95% CI, 39.6–73.9%).

**Fig. 3:**
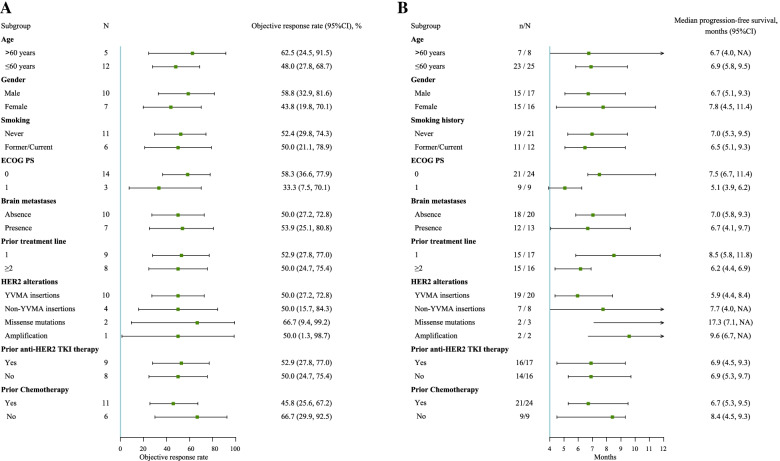
Subgroup analyses of objective response rate (**A**) and progression-free survival (**B**)

In the post hoc subgroup analyses, benefits in terms of ORR and PFS were observed across all subgroups stratified by patient characteristics (Fig. 3). Similar ORRs were observed when pyrotinib combined with apatinib was used in the second-line (52.9%, 9/17; 95% CI, 27.8 to 77.0%) or third- or above-line settings (50.0%, 8/16; 95% CI, 24.7 to 75.4%), and the median PFS times in the second-line therapy group and in the later-line therapy group were 8.5 months (95% CI, 5.8 to 11.8) and 6.2 months (95% CI, 4.4 to 6.9), respectively. In the absence or presence of baseline brain metastases, the ORR was 50.0% (10/20; 95% CI, 27.2 to 72.8%) versus 53.8% (7/13; 95% CI, 25.1 to 80.8%), and the median PFS times were 7.0 (95% CI, 5.8 to 9.3) months and 6.7 (95% CI, 4.1 to 9.7) months, respectively. Among patients with different *HER2* exon 20 insertions, the ORR was 50.0% (95% CI, 27.2 to 72.8%) for those with YVMA insertions and 50.0% (95% CI, 15.7 to 84.3%) for those with non-YVMA insertions; the median PFS times were 5.9 (95% CI, 4.4 to 8.4) months and 7.7 (95% CI, 4.0 to NA) months, respectively.

### Safety

All patients experienced any grade of TRAEs, of whom 12.1% had at least one grade 3 TRAE. As summarized in Table 2, the vast majority of TRAEs were grade 1 or 2, with the most common being diarrhea (90.9%), hypertension (72.7%), anorexia (54.5%), nausea (51.5%), and oral mucositis (45.5%). Grade 3 TRAEs were diarrhea (3.0%) and hypertension (9.1%). No grade 4 or 5 TRAEs were reported. One patient (3.0%) discontinued study treatment because of intolerable vomiting and nausea, and 10 patients (30.3%) experienced pyrotinib dose reduction (320 mg once daily) or dose interruption related to TRAEs, including seven with grade 2 diarrhea and one each with grade 3 diarrhea, grade 2 vomiting, and grade 2 nausea and vomiting. Five patients (15.2%) discontinued apatinib treatment owing to grade 3 hypertension, grade 2 hand-foot syndrome, and grade 2 nausea and vomiting.

**Table 2:** Treatment-related adverse events by pyrotinib combined with apatinib

Event	Patients (*N* = 33)	
	**Any grade, *n* (%)**	**Grade 3, *n* (%)**
Diarrhea	30 (90.9)	1 (3.0)
Hypertension	24 (72.7)	3 (9.1)
Anorexia	18 (54.5)	0 (0.0)
Nausea	17 (51.5)	0 (0.0)
Oral mucositis	15 (45.5)	0 (0.0)
Erythra	13 (39.4)	0 (0.0)
Vomiting	10 (30.3)	0 (0.0)
Hand-foot syndrome	9 (27.3)	0 (0.0)
Abdominal pain	7 (21.2)	0 (0.0)
Blood creatinine increased	6 (18.2)	0 (0.0)
Thrombocytopenia	4 (12.1)	0 (0.0)
Anemia	3 (9.1)	0 (0.0)
ALT increased	3 (9.1)	0 (0.0)
AST increased	3 (9.1)	0 (0.0)
Paronychia	3 (9.1)	0 (0.0)
Leukopenia	2 (6.1)	0 (0.0)
Proteinuria	2 (6.1)	0 (0.0)

*ALT* Alanine aminotransferase, *AST* Aspartate aminotransferase

## Discussion

To our knowledge, this is the first trial to report the efficacy and safety of combination therapy of a *HER2*-targeted TKI and an antiangiogenic agent in patients with *HER2*-altered metastatic NSCLC who have failed at least first-line treatment. This phase 2 trial met its primary endpoint, with pyrotinib combined with apatinib yielding a confirmed ORR of 51.5%. In addition, the DCR was 93.9%, median DoR was 6.0 months, median PFS was 6.9 months and median OS was 14.8 months.

As per previous reports, poor antitumor activity was observed with single-agent afatinib, dacomitinib, and neratinib in advanced NSCLC with *HER2* mutations, with an ORR of only 3.8%-12.0% and a median PFS of 3–5.5 months [7, 9, 10]. Recently, Elamin and colleagues reported that poziotinib resulted in an ORR of 27%, median PFS of 5.5 months and median OS of 15 months in 30 patients with *HER2* exon 20 mutated NSCLC [8]. In patients with advanced *HER2*-mutated lung adenocarcinoma who had received platinum-based chemotherapy, pyrotinib monotherapy showed an ORR of 30.0%, median PFS of 6.9 months and median OS of 14.4 months [22]. Although cross-trial comparisons should be interpreted with caution, our findings suggest that the combination of pyrotinib and apatinib produced a meaningful improvement in ORR.

In addition to *HER2*-targeted TKIs, other promising strategies have been explored in *HER2*-mutated NSCLC. In the MyPathway study, patients with *HER2*-mutated tumors received trastuzumab plus pertuzumab and had an ORR of 21% [25]. In the IFCT-1703 R2D2 trial, triplet trastuzumab, pertuzumab, and docetaxel yielded an ORR of 29% and a median PFS of 6.8 months in patients with advanced NSCLC harboring *HER2* mutations [26]. Additionally, T-DM1 presented an ORR of 44% and a median PFS of 5 months in *HER2*-mutant lung cancers [14]. DS-8201 produced a 55% ORR and median PFS of 8.2 months in patients with pretreated *HER2*-mutated NSCLC [15]. It should be noted that 26% of patients treated with DS-8201 developed adjudicated drug-related interstitial lung disease, and two patients died [15]. Unlike the above targeted agents (trastuzumab, T-DM1 and DS-8201), which are administered intravenously, both pyrotinib and apatinib are orally administered, implying a potential improvement in patient compliance.

**Fig. 4:**
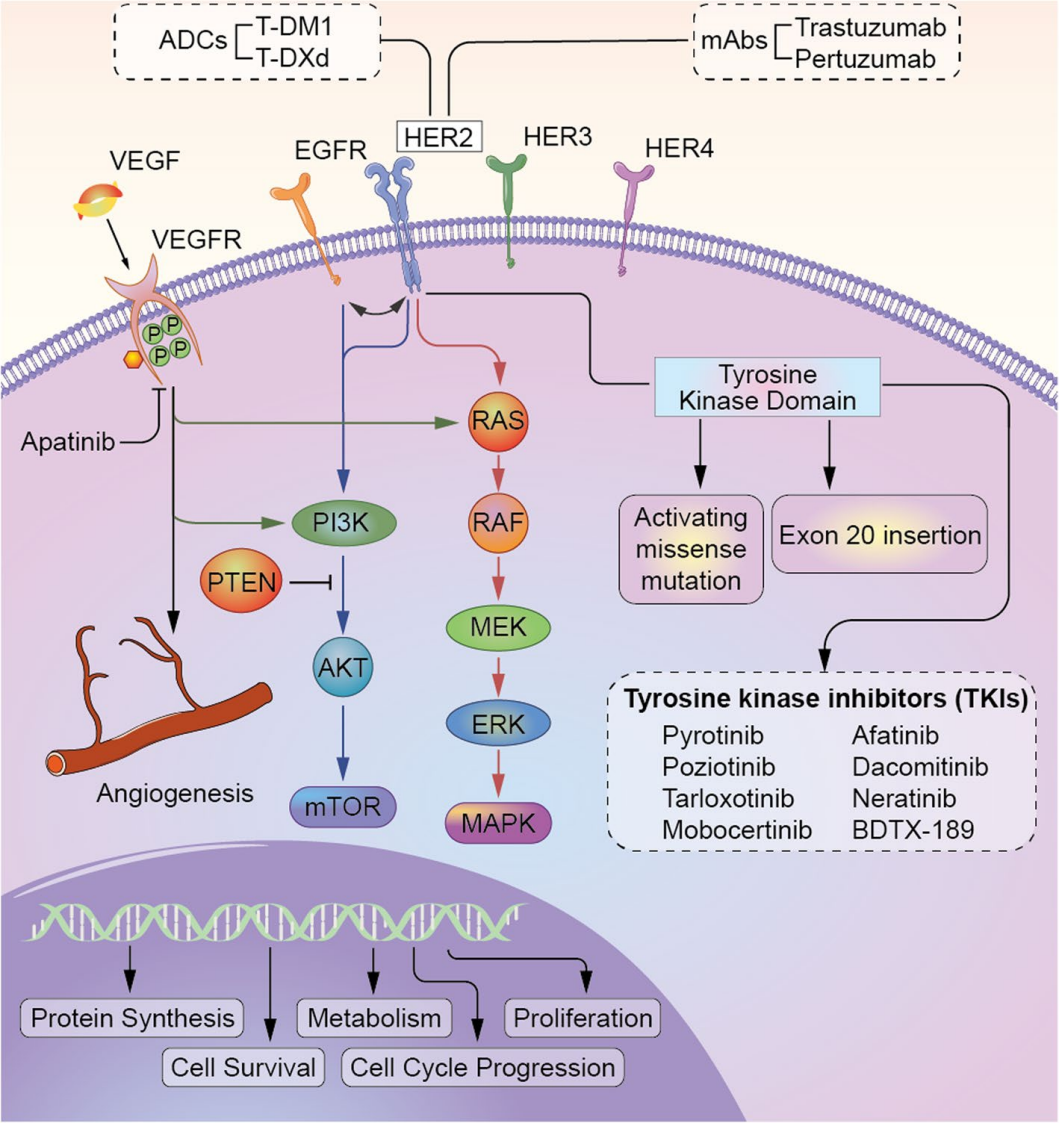
Landscape of *HER2* signaling pathways and mechanistic basis for angiogenesis inhibitor and *HER2*-targeted agents in *HER2*-altered NSCLC

Subgroup analysis showed that comparable responses were observed in patients receiving pyrotinib plus apatinib either as second-line or as third- or above-line therapy, while patients treated with this regimen as second-line therapy had better PFS than those treated with this regimen as third- or above-line therapy (9.8 vs. 6.1 months), which might indicate pyrotinib plus apatinib might be more efficacious in earlier lines for *HER2*-altered NSCLC patients; however, more studies on this topic are needed. The *HER2* receptor is a member of the *ErbB* family of transmembrane tyrosine kinase receptors, and its dimerization catalytically activates its downstream signaling cascades through the *MEK*/*ERK*/*MAPK* and *PI3K*/*AKT*/*mTOR* pathways and mediates tumor angiogenesis by activating the *VEGF* signaling pathway (Fig. 4). This might offer a mechanistic basis for the good efficacy in *HER2*-altered NSCLC patients.

The safety profile of pyrotinib plus apatinib was consistent with that of previous reports for each single agent, without any emerging safety signals identified [22, 27, 28]. The most common TRAEs included diarrhea, hypertension, anorexia, nausea, and oral mucositis, with grades 1–2 being dominant. Four patients experienced grade 3 TRAEs: three experienced hypertension, and one reported diarrhea. All TRAEs were tolerable and well manageable with dose reduction/interruption and symptom-based treatment. Additionally, the low incidence of any-grade proteinuria (6.1%) might be attributed to the low-dose administration of apatinib at 250 mg, which has been proven to be as appropriate and effective as 500 mg and has been applied in numerous clinical studies in NSCLC and other solid tumors [29–31].

Several limitations should be noted in our trial. First, this was a single-center, single-arm, exploratory phase 2 study with a small sample size; thus, selection bias seems unavoidable. Second, only a few patients with *HER2* missense mutations or *HER2* amplification were included, with no confirmatory information on the clinical efficacy of pyrotinib combined with apatinib in this setting.

## Conclusions

Pyrotinib combined with apatinib showed encouraging antitumor activity and an acceptable safety profile in metastatic NSCLC patients with heterogeneous *HER2* mutations or amplification, indicating that it might be a potential effective strategy for *HER2*-mutant or *HER2*-amplified NSCLC.

## Data Availability

The datasets used and/or analyzed during the current study are available from the corresponding author upon reasonable request.

## Abbreviations

ADCs: Antibody-drug conjugates
AEs: Adverse events
ALK: Anaplastic lymphoma kinase
CI: Confidence interval
CNS: Central nervous system
CR: Complete response
CT: Computed tomography
DCR: Disease control rate
DoR: Duration of response
ECOG PS: Eastern Cooperative Oncology Group performance status
EGFR: Epidermal growth factor receptor
HER2: Human epidermal growth factor receptor 2
MRI: Magnetic resonance imaging
NCI-CTCAE: National Cancer Institute-Common Toxicity Criteria for Adverse Events
NGS: Next-generation sequencing
NSCLC: Non-small cell lung cancer
ORR: Objective response rate
OS: Overall survival
PCR: Polymerase chain reaction
PFS: Progression-free survival
PR: Partial response
RECIST 1.1: Response Evaluation Criteria in Solid Tumours, version 1.1
SD: Stable disease
TKD: Tyrosine kinase domain
TKIs: Tyrosine kinase inhibitors
TRAEs: Treatment-related adverse events
VEGF: Vascular endothelial growth factor
VEGFR: Vascular endothelial growth factor receptor
